# Posterior Malleolus: Morphologic Classification, Morphometry, and Clinical Insights

**DOI:** 10.1177/24730114251341900

**Published:** 2025-06-08

**Authors:** Hellen Carvalho Ribeiro, William Paganini Mayer, Jacob Matz, Josemberg da Silva Baptista

**Affiliations:** 1 Department of Morphology, Federal University of Espirito Santo (UFES), Vitoria, ES, Brazil; 2 Laboratory of Applied Morphology (LEMA-UFES), Vitoria, ES, Brazil; 3 Dalhousie Medicine New Brunswick (DMNB), Saint John, NB, Canada; 4 Canada East Foot & Ankle, Saint John, NB, Canada; 5 Horizon Health Network, Saint John, NB, Canada

**Keywords:** ankle joint, tibia, ankle fracture, ankle injury, posterior malleolus fracture

## Abstract

**Background:**

In this study, we provide a comprehensive description of the morphometrics of the distal tibiae and propose that the intact posterior malleolus (PM) exhibits clinically relevant morphologic variation. These differences may have implications for fracture classification, fixation strategy, and implant design.

**Methods:**

Fifty-two isolated dry tibias were analyzed to determine the PM morphometric parameters. Five key morphometric points were identified, and the PM was defined as the posterior bony projection of the distal tibial epiphysis. The malleolar groove established the PM’s medial limitation, the posterior portion of the fibular notch defined the lateral limit, and the anterior boundary was a line connecting these landmarks across the inferior articular surface. PM shapes were categorized based on consistent morphologic patterns. Cross-sections of the distal tibia were performed to assess trabecular bone morphology and density.

**Results:**

We found the PM presenting 3 distinct morphologic types: rounded, triangular, and trapezoid. Triangular and trapezoid types exhibited larger dimensions and robust bone tissue, whereas tibias with a rounded PM displayed smaller dimensions and delicate bone architecture.

**Conclusion:**

These novel findings reveal PM morphologic diversity, which may enhance our understanding of PM fracture patterns and optimize the development of surgical implants.

## Graphical abstract

**Visual Abstract:**
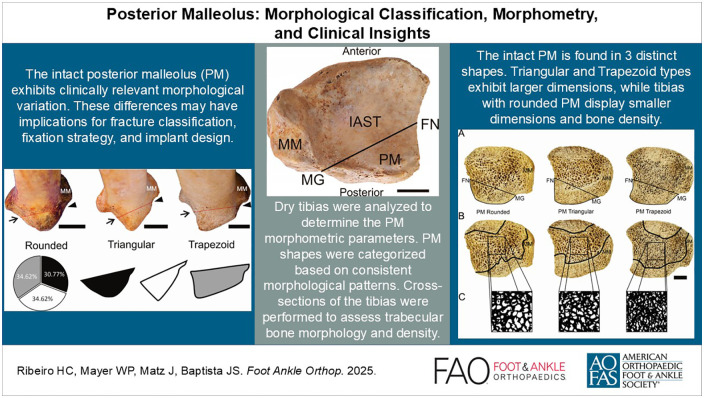
This is a visual representation of the abstract.

## Introduction

The PM is reported to be associated with approximately one-third of ankle joint injuries, and the literature conveys multiple approaches to the treatment of PM injuries.^3,11,23^ The clinical and surgical literature picture the PM with 2 anatomical structures: the posterior tubercle, a posterior projection of the distal epiphysis of the tibia, and the fibular notch (FN; the indentation of the distal epiphysis of the tibia that receives the fibula).^3^

Biomechanical studies demonstrated that the structure of the PM plays a key role in the tibiotalar weightbearing process and posterior stability of the ankle.^3,11,15,23^ The PM contributes to the tibiofibular syndesmotic stability by supporting the attachment of the posterior inferior tibiofibular ligament (PITFL), one of the ligaments composing the tibiofibular syndesmosis. Cadaveric studies describe that the PITFL blends with the tibialis posterior tendon sheath medially. Laterally into the peroneal tendon sheath, the PITFL then splits into 2 separate insertions onto the tibia, featuring oblique and transverse fibres.^12^ The PITFL also attaches to the posterior tubercle of the PM and the posterior surface of the lateral malleolus, and this ligament accounts for 42% of the stability of the tibiofibular syndesmosis.^3,11,12^ Additionally, the PM provides congruence compatible with the superior articular surface of the talus to improve ankle stability and prevent posterior subluxation.^15,23^ Furthermore, the posterior tilt of the PM toward its inferior articular surface restrains posterior linear translation of the talus over the tibia.^3^

In the transition between the medial malleolus (MM) and the PM lies the malleolar groove, also known as the tibial retro-malleolar groove or tibialis posterior groove. The tibialis posterior tendon (TPT) glides through this groove, shaping its morphology. Often, dysfunction or entrapment of the TPT is reported in relation to morphologic changes or fractures of the PM.^1,18^

The operative treatment of PM fractures evolved over the years, and many methods are found in the literature today. In the past, the size of the fractured PM would determine the course of treatment. The so-called “one-third rule” determined that the fragment resulting from the PM fracture should be surgically reduced if greater or equal to one-third of the articular surface of the distal tibia.^11,21^ Although the size of the fragment is a factor that still influences the type of approach, most surgeons choose the treatment based on aspects that offer greater chances of restoring the structural integrity of the ankle.^3,23,24^ Typically, PM fractures are addressed by indirect or direct reduction. In the indirect approach, the PM is not manipulated; the resolution of the fracture is achieved by fixation of the medial and lateral malleolus and PITFL ligamentotaxis. On the other hand, the direct reduction is completed with lag screw or plating techniques fixing the PM in posterolateral or posteromedial orientation.^11,23,24^

Understanding the morphology of the PM is crucial not only for the records of specialized anatomical literature but also for offering important information that may help our understanding of the underlying mechanisms of fractures involving the PM and support the treatment of these injuries. Despite the already recognized importance of the PM in the surgical literature, we could not find specialized anatomical literature regarding its morphologic diversity and bony architecture. Therefore, this work addresses this gap by detailing the morphologic types of the PM, presenting structural morphometric data, and offering clinical insights based on the anatomical findings.

## Methods

### Specimens

**Figure 1.**
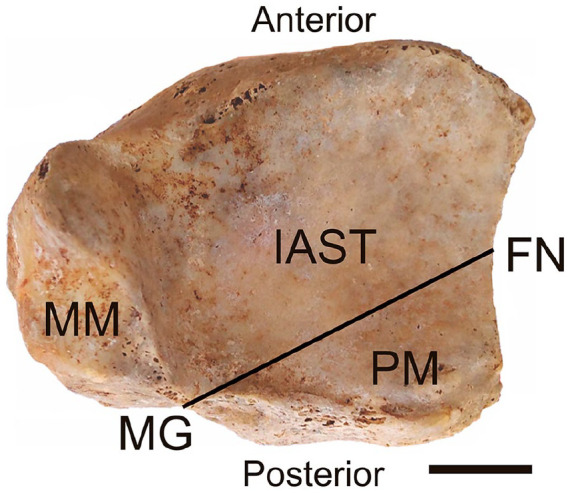
Photograph of the inferior articular surface of the tibia (IAST). The posterior malleolus (PM) is represented by the projection posterior to the line intersecting the malleolar groove (MG) and the midpoint of the fibular notch (FN). Medial malleolus (MM). Scale bar = 2 cm.

The study was conducted on 52 isolated left tibias from an anatomy laboratory collection. The distal epiphysis of the tibias was studied individually to understand the anatomical region and to establish the landmarks of the PM. The following anatomical landmarks related to the PM were considered in this study: the medial malleolus (MM), the malleolar groove (MG), the fibular notch (FN), and the inferior articular surface of the tibia (IAST) (Figure 1). This study was approved by the Institutional Research Ethics Board involving human subjects.

### Morphometry

**Figure 2.**
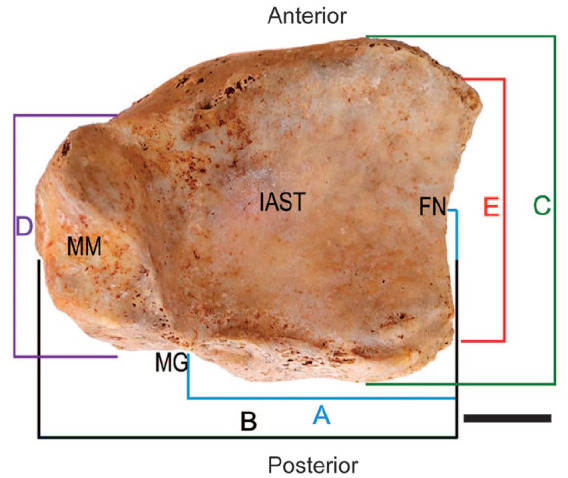
Morphometric points visualized from the inferior articular surface of the tibia (IAST). (A) Length of the posterior malleolus: the distance between the midpoints of the fibular notch (FN) and the malleolar groove (MG). (B) Transverse length of the distal epiphysis: the distance between the surfaces of the FN and the medial malleolus (MM). (C) Anteroposterior length of the distal epiphysis: the distance between the apexes of the anterior and posterior surface of the tibia. (D) Anteroposterior length of the MM: the distance between the anterior and posterior margins of the MM. (E) Anteroposterior length of the FN: the distance between the anterior and posterior margins of the FN. Scale bar = 2 cm.

The PM was defined as the projection of the IAST posterior to a line intersecting the MG and the midpoint of the FN (Figure 1). A digital caliper was used in 5 morphometric points to study the dimensions of the distal tibial epiphysis: the measurement from the MG to the midpoint of the FN represented the length of the PM (Figure 2A); the measurement from the medial surface of the MM to the midpoint of the FN represented the transverse length of the distal epiphysis (Figure 2B); the measurement from the anterior to the posterior edge of the distal tibia represented the anteroposterior length of the distal epiphysis (Figure 2C); the measurement from the anterior edge of the MM to the MG represented the anteroposterior length of the MM (Figure 2D); and the measurement from the anterior tip to the posterior tip of the FN represented the anteroposterior length of the FN (Figure 2E). A single investigator performed all measurements to ensure morphometric systematization and accuracy.

### Morphology and Bone Density

Morphometrics were recorded and listed in the database. A single evaluator characterized the morphologic shape of the PM and categorized the data into groups according to the observed morphology. The various dimensions of the distal tibia were compared among groups to provide morphometric/morphologic insights. We performed cross sections of the distal tibia at the level of the distal epiphyseal line to examine the trabecular bone morphology and density. The cross sections of each morphologic type were photographed and digitized in a Java-based image processing program (ImageJ). Fifteen high-power images with an area of 225 mm^2^ were randomly captured from areas of spongy bone from each cross section and converted to 8-bit grayscale. The optical density of the trabecular bone was defined to determine the gray value threshold. The software calculated the millimeter-to-pixel scale, and the mean gray value (MGV) of the bone in cross section was obtained. The average percentage of trabecular bone in cross sections was expressed over the intertrabecular space in the given area. The estimation of cross-sectional bone density was represented by the pooled average obtained from the mean values from each morphologic type.

### Statistical Analysis

The mean morphometric parameters of each group were calculated, and statistical analysis was performed with the data analysis package for Excel (statistiXL, version 1.8). Measurements were expressed as mean and 95% CI. *F* test was used to verify normal distribution of morphometrics, and 2-sample *t* tests were used to compare morphologic types. Changes were considered statistically significant if *P* < .05.

## Results

### The Posterior Malleolus Displays 3 Morphologic Types: Rounded, Triangular, or Trapezoid

**Figure 3.**
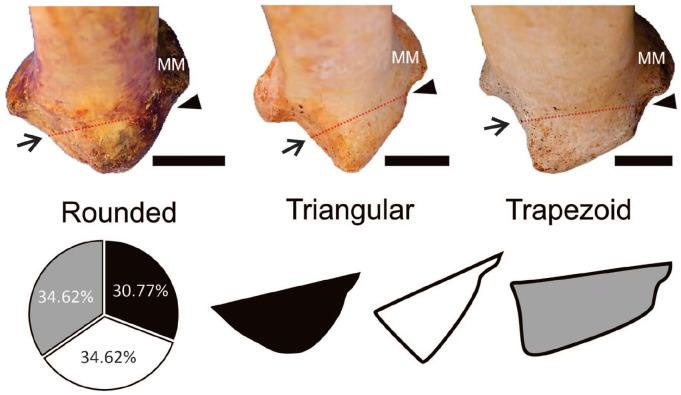
Morphologic classification and incidence of the posterior malleolus types. The posterior malleolus is the structure posterior to the red dashed line intersecting the malleolar groove (arrowhead) and the midpoint of the fibular notch (arrow). Medial malleolus (MM). Scale bar = 3 cm.

The PM was identified as a bony projection of the distal epiphysis of the tibia, posterior to the MG and midpoint of the FN. We observed that the PM exhibited 3 distinct shapes: rounded, triangular, and trapezoidal (Figure 3). n = 16 (30.77%) tibias were classified as rounded, n = 18 (36.62%) as triangular, and n = 18 (36.62%) as trapezoid.

### The Length of the Posterior Malleolus Is Consistent Among the 3 Morphologic Types

**Figure 4.**
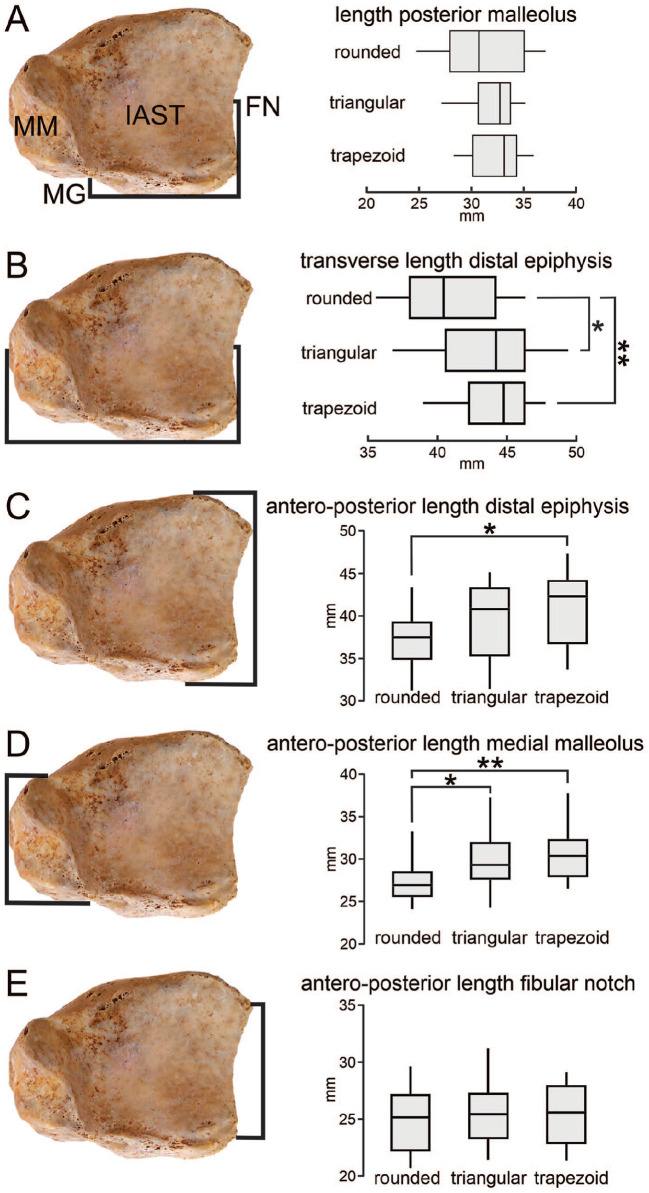
Morphometry of the distal tibial epiphysis. (A) Length of the posterior malleolus. (B) Transverse length of the distal epiphysis. (C) Anteroposterior length of the distal epiphysis. (D) Anteroposterior length of the medial malleolus. (E) Anteroposterior length of the fibular notch. Medial Malleolus (MM). Malleolar groove (MG). Fibular notch (FN). Inferior articular surface of the tibia (IAST). In the box plots, the lower boundary of the box indicates the 25th percentile and the upper boundary of the box the 75th percentile; the black line within the box indicates the median; the pooled average of measurements is represented by the squares and standard deviation by its whiskers. *P < .05; **P < .005.

The distance between the midpoint of the FN and MG represented the length of the posterior malleolus. Rounded PM measured 31.08 mm (95% CI 28.90-33.25), triangular PM 32.14 mm (95% CI 31.00-33.28), and trapezoid 32.47 mm (95% CI 31.25-33.68). The length of the PM did not differ significantly between rounded and triangular (*P* = .367), rounded and trapezoid (*P* = .247), and triangular and trapezoid (*P* = .682) morphologic types (Figure 4A).

### The Distal Epiphysis of Tibias With Rounded Posterior Malleolus Have Narrow Transverse Length

The morphometric points between the MM and FN represented the laterolateral dimensions of the distal tibial epiphysis. This transverse length measured 40.77 mm (95% CI 38.97-42.56) in rounded types, 43.41 mm (95% CI 41.60-45.22) in triangular, and 44.26 mm (95% CI 43.04-45.49) in trapezoid types. The transverse length of tibias with rounded PM was significantly smaller compared with triangular types (*P* = .036) and trapezoid (*P* = .001) types. However, this laterolateral dimension between triangular and trapezoid types did not differ significantly (*P* = .415) (Figure 4B).

### Tibias With Rounded Posterior Malleolus Display Smaller Anteroposterior Dimensions Than Trapezoid Morphologic Types

Distance between the most anterior point of the distal tibial epiphysis and the PM apex provided the anteroposterior length of the distal epiphysis. Tibias with rounded PM types had anteroposterior epiphysial lengths of 37.47 mm (95% CI 35.71-39.22), 39.74 mm (95% CI 37.55-41.92) in triangular, and 40.96 mm (95% CI 38.83-43.10) in trapezoid morphologic types. The anteroposterior length of tibias with rounded PM was significantly smaller compared with trapezoid (*P* = .013) type. This anteroposterior dimension was not significantly different between triangular and trapezoid types (*P* = .404) or between rounded and triangular types (*P* = .102) (Figure 4C).

### Tibias With Rounded Posterior Malleolus Have Smaller Medial Malleolus

Anterior and posterior morphometric points from the MM were investigated among the tibias. The MM of tibias with rounded PM measured 27.46 mm (95% CI 26.15-28.77), 29.62 (95% CI 27.88-31.37) in triangular, and 30.44 mm (95% CI 28.93-31.95) in trapezoid types. Tibias with rounded PM had significantly smaller MM than triangular types (*P* = .048) and trapezoid (*P* = .004) types. Nevertheless, the MM did not differ significantly between triangular and trapezoid types (*P* = .458) (Figure 4D).

### The Fibular Notch Has Similar Dimensions Among Tibias Independent of Its Posterior Malleolus Morphology

The length of the FN was consistent throughout our samples. Tibias with rounded PM had FN with anteroposterior length of 24.87 mm (95% CI 23.36-26.37), triangular PM tibias had 25.27 mm (95% CI 23.93-26.61), and tibias with PM trapezoid presented FN with 25.67 mm (95% CI 24.44-26.91). The length of the FN was not significantly different between rounded and triangular (*P* = .672), rounded and trapezoid (*P* = .383), or triangular and trapezoid (*P* = .646) morphologic types (Figure 4E).

### Tibias With Rounded Posterior Malleolus Have Dissimilar Cortical and Trabecular Bone Architecture

**Figure 5.**
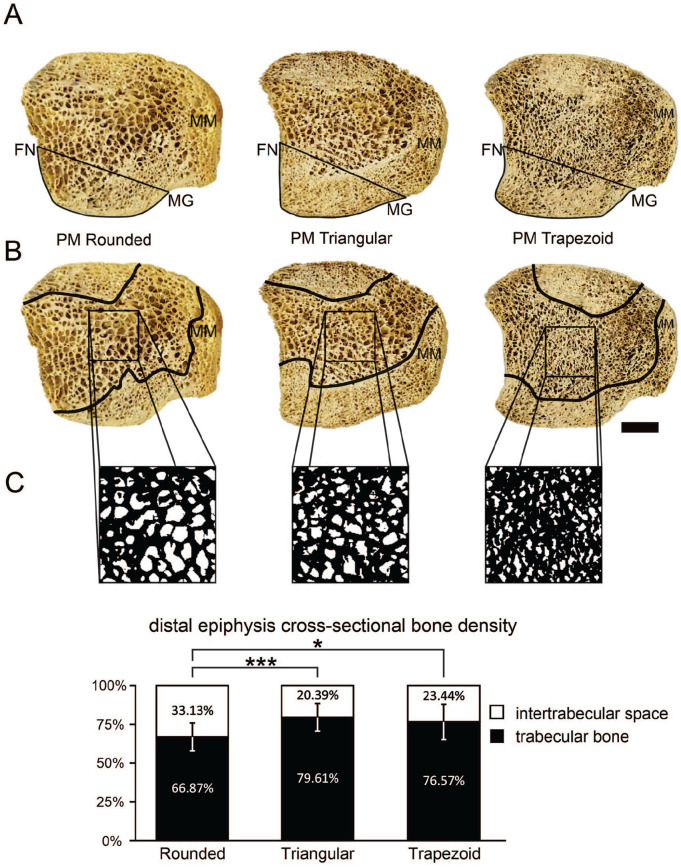
Bone architecture of the posterior malleolus (PM). (A) Cross-sectional morphology of the 3 PM types, (FN) fibular notch, (MG) malleolar groove. (B) Compact and spongy bone morphology of the 3 PM types. Note the thicker and similar compact bone arrangement in triangular and trapezoid PM. (C) Cross-sectional bone density of the tibial distal epiphysis. Insets show the trabecular arrangement of the 3 PM morphologic types, and plots show bone density in percentage. *P < .05; ***P < .000.

The distal epiphysis of the analyzed tibias presented the usual arrangement of peripheral cortical bone and centralized trabecular bone within the epiphysis. Analyzing the region qualitatively, we noticed that the cortical tissue was relatively larger in the PM compared to the anterior and lateral regions of the distal tibial epiphysis independent of the PM morphology. However, we observed that rounded PM exhibited more trabecular bone in its structure, contrasting with triangular and trapezoid types that apparently display more cortical bone in the PM morphology (Figure 5A). In addition, tibias with rounded PM are likely to show thinner and irregular cortical bone, whereas triangular and trapezoid types reveal similar arrangements of their cortical bone structure (Figure 5B).

### Lower Bone Density Is Found in the Distal Epiphysis of Tibias With Rounded Posterior Malleolus

Spongy bone trabeculae were found organized and interconnected, enclosing intertrabecular spaces in circles, hexagons, triangles, or honeycomb-like structures in all PM morphologic types. The density of the spongy bone was greater in both triangular and trapezoid types of PM. Tibias with rounded PM showed cross-sectional bone density of 66.87% (95% CI 61.91-71.86), triangular 79.60% (95% CI 75.42-83.78), and trapezoid 76.56% (95% CI 70.26-82.86). The distal epiphysis of tibias with rounded PM had significantly lower bone density than triangular types (*P* = .000) and trapezoid (*P* = .015) types. Bone density did not differ significantly between tibias with PM triangular and trapezoid (*P* = .382) (Figure 5C).

## Discussion

This study demonstrates that the posterior malleolus exhibits substantial morphologic variation, even in nonpathologic specimens. By analyzing unfractured distal tibias, we identified distinct and reproducible PM shapes that may serve as a basis for future classification systems. We found that the PM is a posterior anatomical projection of the distal epiphysis of the tibia displaying 3 well-differentiated morphologic types. The 3 PM morphologic types were similarly distributed in our samples: 30.77% rounded, 34.62% triangular, and 34.62% trapezoid type. Additionally, tibias with rounded PM displayed smaller distal epiphysis morphometric dimensions. An interesting and novel finding was that the cortical and trabecular bone structure and the bone density of the rounded PM differ from those of triangular and trapezoid morphology. Bone density is often measured using a technique called dual-energy X-ray absorptiometry (DEXA or DXA), and the results are usually expressed in terms of *T* scores. In percentage terms, the rounded PM tibias averaged a bone density of 66.87%, translating to a *T* score of –2.5, which suggests osteoporosis in DEXA scans.^14^ Understanding this variability may also assist in interpreting fracture patterns and guiding preoperative planning.

The distal tibiofibular syndesmosis is known for its important role in stabilizing the ankle joint. The posterior aspect of the FN, a crucial point of attachment for the syndesmoses, contributes to the morphologic configuration of the PM. Although our original and novel findings allow for the characterization of the PM into 3 distinct morphologic shapes, recent research has also classified the tibiofibular syndesmosis in cross-sectional images into rectangular, crescent, and semicircular types.^10^ It is possible that rounded PM are aligned with rectangular cross-sectional tibiofibular syndesmosis whereas triangular and trapezoid PM may associate with crescent and semicircle configurations of tibiofibular syndesmosis respectively. Conceivably, the syndesmosis stability may be related to the morphologic way in which the FN notch and PM interact with the distal end of the fibula to provide optimized fit and ligament insertion.

The TPT lies against the posterior surface of the distal tibia in the malleolar groove. The TPT is the sole tendon in that interface, making it susceptible to injury in cases of distal tibia fractures. Recent studies yield the risk of TPT entrapment in high-energy injuries such as Pilon or PM fractures, especially if the fracture line enters the TPT sheath.^1^ According to our morphologic classification of the PM, it is noticeable that the MG shows variations in depth across different shapes of PM. It is unclear whether the shape of the PM and MG may influence fracture lines and TPT entrapment, and further research is necessary to address these questions.

In the realm of PM fractures, these are commonly framed into the following classifications: Haraguchi (types 1-3),^9^ Mason (types 1, 2A, 2B, and 3),^16^ and Bartoníček (types 1-4).^2^ Among those, Haraguchi type 2, Mason type 2B and 3, and Bartoníček type 3 are more extensive fractures that involve not only the PM but also parts of the MM.^20^ We identified that the rounded PM displayed lower bone density and irregular trabecular bone than other PM types. Although not previously suggested, it is possible that this structural difference may play a role in the orientation of fracture lines, the size, and the number of fragments following an injury.^6,13,17,23,24^ In turn, these factors influence surgical decision making, affecting both the surgical approach and the specific strategy for fixation. PM fractures consisting of multiple fragments require recognition and appropriate reduction to minimize joint incongruity and decrease the propensity for the development of posttraumatic osteoarthritis.^4,24^ These patterns often require the application of plates and screws for stabilization.^17,22^ Precontoured plates are commercially available for many parts of the appendicular skeleton, making fracture fixation more secure, efficient, and more reproducible.^5,7,19^ The recognition of PM morphologic types may lead to more refined hardware options for these fractures.

Our study has limitations. Our sampling was composed of isolated dry tibias from an anatomy laboratory. All the specimens presented fused distal epiphyseal plates, indicating that the samples were from individuals over 16 (female) or 19 (male) years of age.^8^ However, we could not determine sexes or provide further demographics based on isolated tibia analysis. The specimens used in this research enabled us to take direct measurements of distal tibias without additional size information for normalization. Future studies investigating and correlating PM morphology with age, sex, body stature, BMI, or bone mass are important to address these limitations.

The identification of 3 morphologic shapes of the PM and the differences in bone density create questions for future research. Particularly, the influence of PM shape and bone density on the fracture patterns involving the PM is unknown. Furthermore, questions surrounding the syndesmosis relative to PM shape are pertinent, such as stability, propensity for malreduction, ligament morphology, etc. Future clinical studies may be able to address these new questions.

## Conclusion

The PM is found in 3 distinct morphologic shapes, in which the triangular and trapezoid types exhibit larger dimensions. Tibias with rounded PM display smaller dimensions and higher percentage of cancellous bone. Future studies should evaluate whether these morphologic patterns correlate with specific fracture types or clinical outcomes.

## Supplemental Material

sj-pdf-1-fao-10.1177_24730114251341900 – Supplemental material for Posterior Malleolus: Morphologic Classification, Morphometry, and Clinical Insights Supplemental material, sj-pdf-1-fao-10.1177_24730114251341900 for Posterior Malleolus: Morphologic Classification, Morphometry, and Clinical Insights by Hellen Carvalho Ribeiro, William Paganini Mayer, Jacob Matz and Josemberg da Silva Baptista in Foot & Ankle Orthopaedics
